# Prevalence and molecular epidemiology of *Staphylococcus aureus* in Swedish nursing homes – as revealed in the SHADES study†

**DOI:** 10.1017/S0950268813002033

**Published:** 2013-08-20

**Authors:** L. STARK, M. OLOFSSON, S. LÖFGREN, S. MÖLSTAD, P.-E. LINDGREN, A. MATUSSEK

**Affiliations:** 1 Clinical Microbiology, Department of Laboratory Medicine, Ryhov County Hospital, Jönköping; 2 Division of Medical Microbiology, Department of Clinical and Experimental Medicine, Linköping University, Linköping; 3 Ödeshög Health Care Centre, Ödeshög; 4 Division of Community Medicine, Department of Medical and Health Sciences, Linköping University, Linköping; 5 Division of General Practice/Family Medicine, Department of Clinical Sciences in Malmö, Lund University, Malmö

**Keywords:** Colonization, molecular typing, multiclonality, nursing home, *spa* type

## Abstract

Knowledge of carriage and population dynamics of *Staphylococcus aureus* is crucial for infection risk assessment and to reveal transmission patterns of strains. We report the prevalence and molecular epidemiology of *S. aureus* in elderly people (*n* = 290) living in nursing homes in three cities in the south of Sweden. The overall carriage prevalence rate was 48% when results from nares (31%) and throat (34%) samples were combined. Common *spa* types were equally distributed but a frequent type, t160, was found only in one of the regions. Carriage of different *spa* types was detected in 23% of individuals and antimicrobial resistance rates were higher in *S. aureus* isolates from those carrying more than one *spa* type. Five of the 21 individuals who carried different *spa* types were colonized simultaneously with resistant and non-resistant strains. Seventeen per cent of the individuals carried *S. aureus* of the same *spa* type on all occasions. Methicillin resistance was not detected. In conclusion we found a high prevalence of *S. aureus* in this elderly population with a high rate of dual colonization with different *spa* types. We also found signs of institutional spread of one strain.

## INTRODUCTION

Molecular epidemiological studies of *Staphylococcus aureus* have often focused on methicillin-resistant *S. aureus* (MRSA) in outbreak situations. Recent studies indicate that the prevalence of MRSA is low in general populations in European countries [1, 2]. The clinical significance of methicillin-sensitive *S. aureus* (MSSA) is, however, also great [3, 4], and some *spa* types are indicated to be more common in clinical isolates compared to community isolates [5]. Differences in the distribution of MSSA and MRSA strain types have been reported [6] and show a greater diversity of *spa* types in MSSA [2, 5–7], compared to the often observed clonal spread of MRSA strain lineages [7, 8].

An association of specific MSSA *spa* types with age and gender in the general population has been suggested [2]. Moreover, nursing homes have been implicated as reservoirs for antimicrobial-resistant organisms, including *S. aureus* [9]. Only a few comprehensive *S. aureus* screening studies have been performed within these settings, and most focused primarily on MRSA [10, 11].

The overall prevalence of nasal *S. aureus* colonization varies in different studies, and rates up to 55% have been reported [12]. Carriers are generally classified as persistent (∼20%), intermittent (∼30%) and non-carriers (∼50%) [13, 14]. The classification of an individual as a persistent carrier is of importance, since they appear to have an increased risk of endogenous infection compared to non-carriers [15, 16], whereas intermittent carriers have an equally low risk as non-carriers [17]. However, it should be noted that classification of a persistent carriage state remains contentious [18].

Recent studies have shown an equal or higher colonization rate of *S. aureus* in the throat compared to nares, and there are a number of individuals exclusively colonized in the throat [13, 19, 20]. A possible role of throat carriage for *S. aureus* transmission has also been recently indicated [21], and inclusion of throat culture in screening protocols is widely considered to be necessary for the optimal recovery of *S. aureus* [20, 22, 23].

Knowledge of molecular epidemiology of *S. aureus* in nursing-home residents is important as this group represents a population with high comorbidity and frailty [24]. Furthermore, they have an increased frequency of hospital care with a risk of exchange of bacterial strains [9], which might have an influence on transmission patterns. The aim of this study was to determine the prevalence of *S. aureus* in Swedish nursing-home residents and investigate the molecular epidemiology of isolates in terms of the spatial and temporal *spa* gene type distribution and antimicrobial resistance.

## METHODS

### Study population

The study population (*n* = 290; 70% women, 30% men) was recruited from individuals, between the ages of 60 and 101 (median 85) years, included in the SHADES programme [24], a longitudinal, open cohort, multi-purpose study conducted in Swedish nursing homes for permanent residents, between 2008 and 2011. The nursing homes (*n* = 9) were located in three cities in the south of Sweden, Eslöv (*n* = 2) with 47% of the participants, Jönköping (*n* = 4) with 41% of the participants and Linköping (*n* = 3) with 11% of the participants. The colonization rates of *S. aureus* and antibiotic susceptibility results from the first culture have in part been reported previously [23]. Information about age, gender and length of stay were registered. The study was approved by the Regional Ethical Review Board at Linköping University (M150-07). Informed consent was obtained from all included individuals (or from persons close to them as appropriate).

### Sample collection and *S. aureus* isolation and cultivation

Sampling was performed using a rayon-tipped swab (Copan Diagnostics Inc., Italy), from 1 January 2009 to 31 March 2011 and included samples (*n* = 1860) from the anterior nares, the throat, the groin and active skin lesions. If possible, samples were collected every 6 months during the study period.

The swabs were incubated for 16–20 h in a broth selective for *S. aureus* [13] and then 10 μl broth was cultured on blood agar plates (*n* = 996), and from 1 February 2010 on BBL™ CHROMagar™ Staph aureus medium (Becton Dickinson, USA) (*n* = 864). Plates were incubated for 20–24 h, and *S. aureus* was confirmed by DNase activity or detection of the *nuc* gene [25]. Isolates were tested for antibiotic susceptibility [23] and stored at –80°C in skimmed milk.

### *spa* typing

All *S. aureus* isolates (*n* = 466) were *spa* typed according to Kahl *et al*. [26]. PCR products were sequenced at GATC Biotech (GATC Biotech AG, Germany). Ridom StaphType software (Ridom GmbH, Germany) was used to determine the *spa* types [27] and BURP clustering was performed [28].

### Statistical analyses

Statistical analysis (*χ*^2^ test and *t* test) was performed using the IBM SPSS Statistics version 20 statistical package (IBM Corporation, USA) and included the prevalence of colonization with *S. aureus* at the four body sites, the proportion of antibiotic-resistant isolates and the occurrence of *spa* types specified by individual characteristics and geographical region.

## RESULTS

### Prevalence

On the first sampling occasion 179/290 individuals were sampled from nares, throat and groin. From the remaining 111 subjects only samples from one or two of the body sites were available. There were no differences in age, gender and length of stay in the nursing home between individuals sampled on three body sites compared to those sampled from one or two body sites. Overall, 185 isolates of *S. aureus* from 747 samples were obtained. Table 1 shows that the highest prevalence was found in throat samples (34%) followed by anterior nares (31%), and by combining nares and throat samples the prevalence increased to 48%. There was no significant difference in gender (*P* = 0·52) or mean age (*P* = 0·14) between individuals colonized and not colonized.

**Table 1.** Detection of *S. aureus* and *spa*-type distribution, in different body sites, at the first sampling occasion

	Body site					
	Nares	Throat	Groin	Skin lesion	Nares and throat	Nares, throat and groin
Number of individuals sampled	285	180	245	17	179	179
Individuals from whom *S. aureus* was isolated, *n* (%)	87 (31)	62 (34)	24 (10)	7 (41)	86 (48)*	88 (49)*
Number of different *spa* types	59	43	15	7		
Most common *spa* types found in different individuals (%)‡	t002 (6·8)	t002 (8·2)	t002 (16·7)	t160†, t002, t008, t015, t164, t505, t9208		
	t160 (5·7)	t015 (6·6)	t1040 (8·3)			
	t015 (5·7)	t084 (6·6)	t160 (8·3)			
	t295 (3·4)	t008 (4·9)	t709 (8·3)			
	t008 (3·4)	t065 (4·9) t709 (4·9)	t728 (8·3)			

* *S. aureus* detected in one or more of the body sites.

† *spa* type t160 was found in two individuals, the rest in one each.

‡ The proportion of individuals carrying the *spa* type at the given body site.

**Fig. 1.**
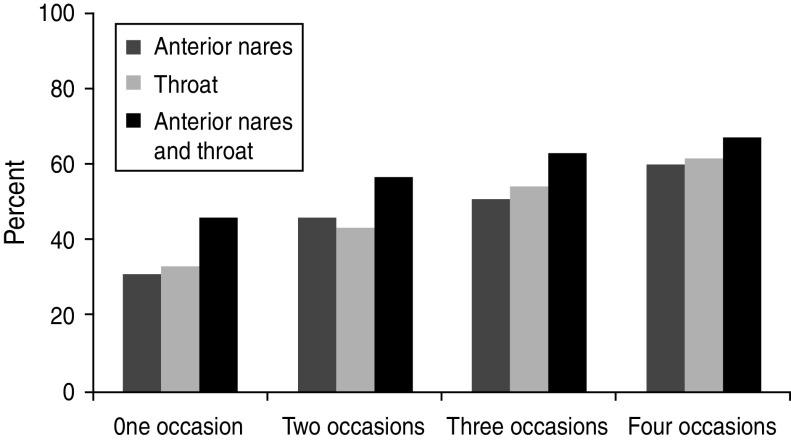
The prevalence of *S. aureus* colonization rates obtained by the accumulation of results from increasing sampling occasions.

In the longitudinal study the 290 individuals were sampled on various occasions (*n* = 1–5) for up to 2 years, due to the open cohort design; 28% once, 21% twice, 18% three times, 28% four times and 5% five times. A total of 1860 samples was obtained; nares (*n* = 739), throat (*n* = 355), groin (*n* = 665) and skin lesions (*n* = 101) and *S. aureus* was isolated from 466 of these cultures: nares (*n* = 240), throat (*n* = 110), groin (*n* = 63) and skin lesions (*n* = 53). The accumulated proportion of individuals from whom *S. aureus* could be isolated, increased from 31% to 60% and from 34% to 62% in nares and throat, respectively, by increasing the number of sampling occasions (*n* = 4), and by combining results from nares and throat the proportion increased to 67% (Fig. 1).

### *spa* type distribution

Of the 185 isolates from the first sampling occasion 73 different *spa* types were identified; 47 (64%) were only found in single individuals, 14 (19%) in two, and 13 (18%) in three or more individuals. One isolate was non-typable. The most common *spa* types were t002 (8·3%), t160 (5·8%), t015 (5·0%), t084 (5·0%) and t705 (3·3%). No difference in distribution of common *spa* types was seen between the nares and throat samples (Table 1). All isolates from the groin and skin lesions belonged to *spa* types also isolated from other body sites. Carriage of two different *spa* types in one individual, was found in 10/43 (23%) individuals from whom *S. aureus* was isolated from more than one body site on the same occasion.

**Fig. 2.**
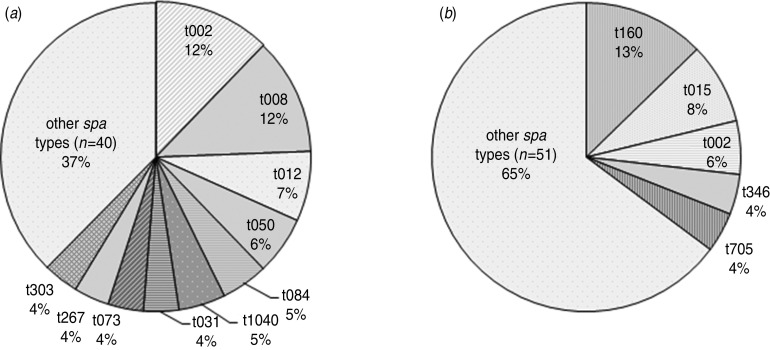
Geographical distribution of *spa* types isolated from more than two individuals in the longitudinal study in (*a*) Eslöv and (*b*) Jönköping.

In the longitudinal study, no temporal differences in the distribution of major *spa* types were seen (data not shown). Fifty different *spa* types distributed over 82 individuals were detected in Eslöv, and in Jönköping 56 different *spa* types in 71 individuals were found. No *spa* type was carried by more than two individuals in Linköping. *S. aureus* of *spa* type t160 was found in nine individuals in one region, but was not found in the other geographical regions whereas other common *spa* types were equally distributed (Fig. 2). Seven of the individuals with *spa* type t160 were living at the same nursing home.

BURP cluster analysis of all *spa* types (*n* = 98) found in the longitudinal study revealed 10 clusters and 14 singletons (seven *spa* types shorter than five repeats were excluded from the analysis). The largest cluster was *spa*-CC 015 including 17% of all *spa* types followed by *spa*-CC 012 (15% of all *spa* types), *spa*-CC 084 (9% of all *spa* types) and *spa*-CC 005 (4% of all *spa* types). Of the 35 individuals that carried different *spa* types at any time during the study period, 21 carried the different strains at the same occasion and 18 changed *spa* type over time. Three individuals carried *spa* types closely related and in two of these individuals both *spa* types were present on the same sampling occasion.

### Carriage of *S. aureus* over time

Carriage over time was evaluated in individuals sampled on three occasions (every 6 months) for 1 year. *S. aureus* was isolated from nares in 20% of the individuals, and 17% carried strains of the same *spa* types over the sampling period (Table 2). Similarly, 7% of the individuals exhibited throat carriage and all carried the same *spa* type. The proportion of individuals from whom *S. aureus* was never isolated was 49% for nares and 46% for throat samples. There was no significant difference in gender (*P* = 0·40), mean age (*P* = 0·40), or length of stay (*P* = 0·69) between those that always carried *S. aureus* and others. Carriage rates based on two sampling occasions within 6 months from nares (*n* = 216) showed *S. aureus* was present in 22% of the individuals, and 19% carried strains of the same *spa* type. *S. aureus* was not detected in 55% of the individuals sampled twice from the nares.

**Table 2.** Repeated detection of *S. aureus* in individuals sampled on three occasions during one year

	Anterior nares (*n* = 144)		Throat (*n* = 46)		Any of three sampling sites (*n* = 44)*	
	Number of individuals (%)	Individuals with the same *spa* type *n* (%)	Number of individuals (%)	Individuals with the same *spa* type *n* (%)	Number of individuals (%)	Individuals with the same *spa* type *n* (%)
*S. aureus* detected on all occasions	29 (20)	24 (17)	3 (7)	3 (7)	11 (25)	9 (20)
*S. aureus* detected on two occasions	15 (10)	11 (8)	8 (17)	6 (30)	9 (20)	6 (14)
*S. aureus* detected on one occasion	29 (20)		14 (30)		11 (25)	
*S. aureus* not detected	71 (49)		21 (46)		13 (30)	

* Individuals sampled from nares, throat and groin on three occasions.

### Antibiotic susceptibility testing

From the 290 individuals in the longitudinal study 338 isolates were available for antibiotic susceptibility testing. Of these 44 isolates from 26 (14 male, 12 female) individuals all but one were resistant to a single antibiotic and the other to two antibiotics (ciprofloxacin and fusidic acid). Overall, 18% of the colonized individuals carried an antibiotic-resistant isolate. None of the isolates was resistant to vancomycin, gentamicin, erythromycin, linezolid or rifampicin and no MRSA was detected. No specific *spa* type was associated with resistance. The proportion of individuals with resistant isolates was higher in the individuals colonized with two different strains compared to carriers with a single clone (*P* = 0·04). Five individuals who carried two different strains were simultaneously colonized with resistant and non-resistant strains.

## DISCUSSION

In this study we investigated the molecular epidemiology of *S. aureus* in a population of the elderly living in nine nursing homes in the south of Sweden. We found a diverse distribution of *spa* types as well as a local spread of one *spa* type. As much as 23% of individuals were colonized simultaneously with isolates of two different *spa* types and in this group antimicrobial resistance was twice as common as for those colonized with a single strain, and five individuals simultaneously carried resistant and susceptible strains. Furthermore, we show that inclusion of throat sampling with nasal swabs as well as repeated sampling increases the sensitivity of *S. aureus* detection in screening programmes.

The high diversity and the most frequently identified *spa* types in our study are in agreement with previous data from general populations as well as from hospital settings [1, 2, 5–7, 29]. The four most common *spa* types were also the most prevalent in the Swedish HITS study, which was conducted in the regions of Jönköping and Linköping, but the fifth most common *spa* type was restricted to the region of Eslöv. This indicates a comparable distribution of *spa* types between regions and populations and suggests that some strains are more successful colonizers than others, as exemplified by the finding of *spa* type t160 in a single nursing home. There are conflicting results in the literature on *spa*-type distribution regarding age and gender [2, 13] but we found no differences in these parameters and body site sampled in our study population.

BURP clustering analysis revealed that only 3/35 individuals carrying different *spa* types at any time were carriers of closely related strains and that almost half of all *spa* types fell into the four largest clusters. The high rate (23%) of individuals harbouring simultaneously two strain types in two or more body sites on a single sampling occasion is noteworthy as only one isolate from each site was subjected to *spa* typing. A previous study calculated the rate of individuals carrying two different strains of *S. aureus* to be below 7%, based on typing of three colonies from the anterior nares [30]. More recently a study in children reported a similar rate (26%) to that found here when 4–15 colonies were typed [31]. The issue of simultaneous carriage of more than one strain is of clinical importance as a more resistant *S. aureus* strain may remain undetected impairing antibiotic treatment, a situation underlined by the recovery of resistant and susceptible phenotypes of different *spa* types from the same individual, albeit from different body sites. Further studies are required to elucidate the clinical significance of these findings. Furthermore, in epidemiological and transmission studies undetected carriage of multiple strains could lead to misinterpretation of data. However, analyses of dual colonization with different strains based on selecting several colonies from agar plates would be laborious and simplified methods are needed [32].

The highest prevalence of *S. aureus* carriage was found in the throat (34%), which is in accordance with a previous Swedish study [13]; the nasal colonization rate of 31% is also comparably high, but in agreement with previous results from a Swedish population [1]. Screening protocols for *S. aureus* carriage based on nares sampling only may underestimate colonization rates. We were able to increase the recovery rate from 31% to 48% by combining the culture results obtained from the nares and the throat on one sampling occasion which has also been shown by other studies [20–22]. In addition, repeated sampling occasions further increased the prevalence figures. Thus the use of a sensitive strategy of multiple body-site sampling on different occasions maximized the estimates of the prevalence of *S. aureus* colonization in this elderly population.

The issue of persistent carriage of *S. aureus* is important, since persistent nasal carriers appear to have an increased risk for endogenous infection [15–17]. There is no general consensus on the definition of persistent carriage [14, 18], and reported frequencies from nasal samples vary between 12% and 30% [12, 13, 17, 33–35]. We found 20% of carriers were repeatedly colonized in the nares on three sampling occasions over 1 year and this rose to 22% if the result was based on two sampling occasions. The importance of persistent throat carriage, as a risk for endogenous infections and in transmission to others has not been fully evaluated. Recently, a persistent throat carrier rate of 46% was reported using enrichment broth prior to plating [13]. These are higher figures than the rates of 24% and 7% repeatedly colonized in the throat on two and three occasions, respectively, found in our study. However, we only succeeded in obtaining three throat samples from 46 individuals due to sampling difficulties.

When persistent carriage is to be defined, the type identity of the strain of *S. aureus* should be considered [13]. It is not known if the risk inherent in being a persistent carrier is dependent on continuously carrying the same strain. We found that 17% of the elderly were colonized in the nares with *S. aureus* of the same *spa* type on all three occasions compared to 20% for all *S. aureus* strain types and the corresponding figures for two sampling occasions were 19% and 22%, respectively.

*S. aureus* was never detected in almost half of nasal (49%) and throat (47%) swabs of participants and they could thus be regarded as non-carriers, which is in agreement with other findings [12, 14, 17, 33–35]. The basis for the resistance of individuals to colonization is not known, but some *spa* types seem to be more successful colonizers of humans [36], and little is known about bacterial factors of importance for specific strains to become virulent [37].

In conclusion, we found a high prevalence of *S. aureus* in this elderly population and a local accumulation of one strain was noted in one nursing home. Antibiotic resistance was rare and no MRSA was found, but in individuals colonized with two different strains resistance was higher and simultaneous detection of resistant and non-resistant strains was registered.
